# The Relationship between Serum Zinc Level and Preeclampsia: A Meta-Analysis

**DOI:** 10.3390/nu7095366

**Published:** 2015-09-15

**Authors:** Yue Ma, Xiaoli Shen, Dongfeng Zhang

**Affiliations:** Department of epidemiology and health statistics, Medical college, Qingdao University, No. 38 Dengzhou Road, Qingdao 266021, China; E-Mails: murphy.ma@163.com (Y.M.); shenxiaoli2000@163.com (X.S.)

**Keywords:** zinc, Zn, preeclampsia, meta-analysis

## Abstract

The association between serum zinc level and preeclampsia (PE) remains controversial. A systematic literature search was performed in PubMed, Web of Science and Embase for relevant available articles. The articles were limited to those in English from January 1990 to April 2015. Observational studies evaluating the association between serum zinc level and PE were included. The *I*^2^ was used to assess heterogeneity and the random effect model (REM) was adopted as the pooling method. The pooled standard mean difference (SMD) with 95% confidence interval (CI) was used to estimate the association between serum zinc level and PE. Seventeen observational studies were included. Compared with healthy pregnancy controls, PE patients have lower serum zinc level in 14 studies about total PE (SMD (95% CI): −0.587 (−0.963, −0.212), Z = 3.06, *p* for Z = 0.002; *I*^2^ = 88.4%, *p* for *I*^2^ < 0.0001). In subgroup analysis, a lower serum zinc level in PE patients compared with healthy pregnancy controls was observed in studies conducted in Asia, studies with zinc level measured in serum, and studies involving fasting participants. The SMD did not differ significantly between studies with healthy pregnancy controls matched by individual age (yes or no), and by individual gestational age (yes or no), respectively. Results from this meta-analysis indicate that serum zinc level in PE patients is significantly lower than that in healthy pregnancy controls. A moderate amount of zinc supplementation during pregnancy is advocated to reduce the incidence of PE.

## 1. Introduction

Preeclampsia (PE) is a progressive, multisystemic disorder developing after 20 weeks of gestation in women with previously normal blood pressure. PE is a syndrome defined by hypertension (blood pressure of 140 mmHg systolic or higher or 90 mmHg diastolic or higher) and proteinuria [1]. The incidence of PE in pregnancies ranges from 2% to 8% in the world [2,3,4]. The World Health Organization reported that PE is a major reason of mother and fetus morbidity and mortality [5]. The complications of PE are the third leading cause of pregnancy-related deaths [6,7].

PE is caused by multiple factors, and some studies have indicated that PE is associated with an imbalance of increased lipid peroxides (LPO) and decreased antioxidants [8,9]. As an antioxidant trace metal, zinc deficiency may cause increasing lipid peroxidation [10]. Many studies attempted to explore the relationship between the changes of serum zinc level in pregnant women and PE, but the results were conflicting. Some studies had discovered significantly lower levels of serum zinc in PE patients than in the control group [11,12,13]. However, other studies found mean serum level of zinc was significantly higher in PE patients than in healthy pregnancy controls [14,15,16]. Meanwhile, some studies found that the serum zinc concentrations were not significantly different between the PE patients and the healthy pregnancy controls [17,18,19]. Therefore, we performed this meta-analysis to assess the relationship between serum zinc level and PE.

## 2. Materials and Methods

We referred to Preferred Reporting Items for Systematic Review and Meta-Analyses (PRISMA) guidelines for reporting of meta analyses [20].

### 2.1. Literature Search and Selection

We performed a systematic literature search from January 1990 to April 2015 using the databases of PubMed, Web of Science and Embase literature databases. The following search terms “zinc”, “Zn” and “preeclampsia” were used to search the English related articles without other restrictions. Moreover, we also reviewed the references of the included studies and review articles to identify additional studies which were not captured by our database searches.

### 2.2. Inclusion and Exclusion Criteria

The inclusion criteria were as follows: (1) Observational study designs; (2) Diagnosis of PE patients was in accordance with the criteria of the American College of Obstetricians and Gynecologists (ACOG) [1]; (3) The blood sample was venous blood and the time of blood collection was medium or late pregnancy; (4) Data of the serum zinc level was available in the results and the data were presented as mean ± standard deviation (SD); (5) Serum zinc level were detected by using atomic absorption spectrometry; (6) The controls were healthy pregnancy controls. We excluded the studies if the data of serum zinc level was higher than 10 times of normalhigh limit [21]. If the units of measurement were not given, the study was also excluded.

All identified studies were carefully reviewed independently by two investigators to determine whether an individual study was eligible for inclusion criteria in this meta-analysis.

### 2.3. Data Extraction

The following data were extracted: the mean ± SD on serum zinc level, first author’s name, publication year, country where the study was performed, mean age, mean gestational age, sample size, whether the participants were fasting or not, the matching of potential confounders and other information. The data of different groups according to the illness severity were also extracted. If the standard error mean (SEM) of zinc level was given in the study, the SD is calculated by the following formula: *SEM* = *SD*/$\sqrt{n}$. Because the underlying units of measurement varied among studies, all units were converted to μmol/L [21].

Data were extracted independently by two investigators with disagreements resolved through discussion. The Newcastle-Ottawa quality assessment scale was used to assess the study quality [22].

### 2.4. Statistical Analysis

All statistical analyses were performed using standard mean difference (SMD) with 95% CI to assess the strength of association between serum zinc level and PE. The SMD is the ratio of the mean difference to the pooled standard deviation. The *I*^2^ was used to assess heterogeneity and the random effect model (REM) was adopted to calculate the pooled SMD. Meta-regression was performed to assess the potentially important covariates that might exert substantial impacts on between-study heterogeneity.

An influence analysis was performed with one study removed at a time to assess whether the results could be affected markedly by a single study. Small-study effect was investigated with funnel plot and Egger test. All statistical analyses were performed with Stata 12.0 (Stata Corporation, College Station, TX, USA). All reported probabilities (*p* values) were two-sided with *p* < 0.05 considered statistically significant.

## 3. Results

### 3.1. Characteristics of Studies

**Figure 1:**
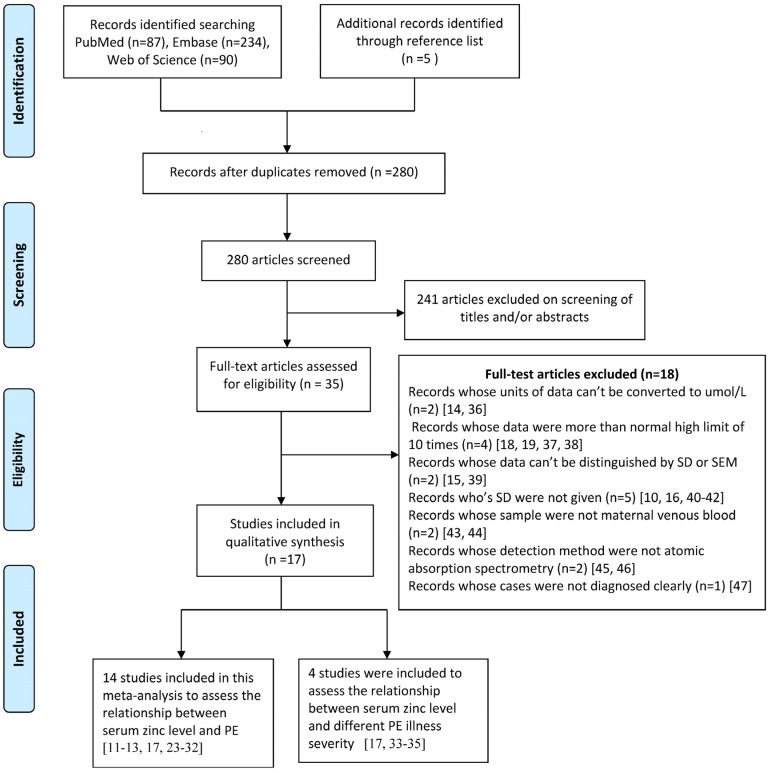
Flow diagram of the literature search.

The detailed steps of our literature search were shown in Figure 1. We identified 17 relevant articles [11,12,13,17,23,24,25,26,27,28,29,30,31,32,33,34,35] in this meta-analysis. There were 3 case-control studies [11,33,34] and 14 cross-sectional studies. The pooled subjects included a total of 725 healthy pregnancy controls and 700 PE cases. Thirteen articles [11,12,13,23,24,25,26,27,28,29,30,31,32] reported the results for total PE (without information for disease severity), and 3 articles [33,34,35] reported the results for mild PE and severe PE, and 1 article [17] reported the results for total PE, mild PE and severe PE. The study quality ranged from 7 stars (5 articles) to 8 stars (12 articles) (Table S1). The characteristics of included articles are shown in Table 1.

**Table 1:** Characteristics of 17 including studies.

Author [Ref.] (Year)	Country Continent	Study Design	Group	*n*	Mean Level of Serum Zinc (μmol/L)	SD (μmol/L)	*p*	Sample Type	Fasting	Age (Years, Mean ± SD)	Gestational Age (Weeks, Mean ± SD)	Match of Potential Confounders
Sarwar, M.S.; [11] (2013)	Bangladesh (Asia)	case-control study	Control PE	58 50	15.08 11.85	3.54 5.38	*p* < 0.001	serum	8 h fasting condition	25.76 ± 0.73 25.46 ± 0.85	36.79 ± 0.27 35.32 ± 0.37	matching for gestational period
Rafeeinia, A.; [17] (2014)	Iran (Asia)	cross-sectional study	Control total PE control mild PE sever PE	50 50 50 35 15	11.23 10.92 11.08 10.62 12.00	5.08 4.00 0.62 0.62 1.23	*p* = 0.76 *p* = 0.71	serum	overnight fast	27.10 ± 4.6 26.50 ± 3.9 27.18 ± 4.6 27.0 ± 4.1 25.40 ± 3.5	31.50 ± 3.60 30.80 ± 3.30 26.41 ± 5.0 23.82 ± 4.60 28.02 ± 7.90	
Fenzl, V.; [24] (2013)	Croatia (Europe)	cross-sectional study	Control PE	37 30	8.85 9.23	1.43 1.43	NS	serum	overnight fast	30.8 31.2	37.42 36.55	
Farzin, L.; [13] (2012)	Iran (Asia)	cross-sectional study	Control PE	60 60	15.48 11.77	3.10 2.71	*p* < 0.001	serum	yes	26.66 ± 3.72 27.43 ± 3.91	35.27 ± 1.20 35.48 ± 1.14	matched for age, gestational age, anthropometrics and socioeconomic status
Adam, B.; [29] (2001)	Turkey (Asia)	cross-sectional study	Control PE	20 20	5.25 4.82	0.68 0.72	NS	plasma	no	27 ± 6.8 29 ± 8	37 ± 3.9 35 ± 4	matched for age, gestational age
Ilhan, N.; [12] (2002)	Turkey (Asia)	cross-sectional study	Control PE	30 21	19.26 12.76	3.73 4.45	*p* < 0.001	plasma	overnight fast	19–31	31–38	
Kolusari, A.; [28] (2008)	Turkey (Asia)	cross-sectional study	Control PE	48 47	0.20 0.16	0.06 0.07	NS	serum	overnight fast	27.92 ± 4.25 27.91 ± 5.21	35.41 ± 1.62 34.87 ± 2.34	
Atamer, Y.; [27] (2005)	Turkey (Asia)	cross-sectional study	Control PE	28 32	16.71 12.18	3.06 2.77	NS	serum	overnight fast	25.85 ± 3.36 27.00 ± 3.89	36.53 ± 3.15 35.68 ± 2.94	
Borella, P.; [30] (1990)	Italy (Europe)	cross-sectional study	Control PE	35 24	9.60 10.49	2.29 2.28	NS	plasma	yes		29–40	
Akhtar, S.; [31] (2011)	Bangladesh (Asia)	cross-sectional study	Control PE	30 60	17.74 13.88	1.03 2.42	*p* < 0.001	serum	no	25.20 ± 4.85 25.11 ± 5.66	31.53 ± 3.90 32.35 ± 3.53	ageand gestational period matched
Akinloye, O.; [23] (2010)	Nigeria (Africa)	cross-sectional study	Control PE	40 49	9.40 8.60	0.80 1.40	*p* < 0.05	serum	no			age-matched
Ahsan, T.; [25] (2013)	Bangladesh (Asia)	cross-sectional study	Control PE	27 44	15.00 16.00	2.00 2.00	*p* = 0.560	serum	no	24.11 ± 4.93 26.05 ± 5.41	36.23 ± 2.64 35.60 ± 3.85	demographically well matched
Rathore, S.; [26] (2011)	India (Asia)	cross-sectional study	Control PE	47 14	8.85 7.57	3.32 2.74	NS	serum	no	19–35		age-matched
Ugwuja, E.I.; [32] (2010)	Nigeria (Africa)	cross-sectional study	Control PE	40 40	10.87 9.97	10.30 9.74	*p* = 0.686	plasma	no	27.55 ± 4.23 29.45 ± 3.70	21.40 ± 3.22	matched for age, gestational age, parity, anthropometrics andsocioeconomic status
Gupta, S.; [33] (2014)	India (Asia)	case-control study	Control mild PE sever PE	75 47 18	10.63 10.46 9.28	1.82 2.05 1.63	NS *p* < 0.01	plasma	no			
Araujo Brito, J.; [34] (2013)	Brazil (America)	case-control study	Control mild PE sever PE	50 20 24	7.43 7.69 5.97	1.28 1.45 1.26	NS *p* < 0.05	plasma	fasting for at least 12 h	24.13 ± 6.43 27.00 ± 6.59	39.17 ± 1.76 36.30 ± 3.01	
Jain, S.; [35] (2010)	India (Asia)	cross-sectional study	Control mild PE sever PE	50 25 25	15.64 12.72 12.04	2.40 1.70 1.40	*p* < 0.05 *p* < 0.05	serum	no	23.92 ± 3.42 23.04 ± 3.76 22.96 ± 3.81	33.62 ± 7.83 34.92 ± 3.54 35.08 ± 3.60	age-matched

Abbreviations: SD: standard deviation; PE: preeclampsia; NS: nosignificant.

### 3.2. Serum or Plasma Zinc Level and PE

**Figure 2:**
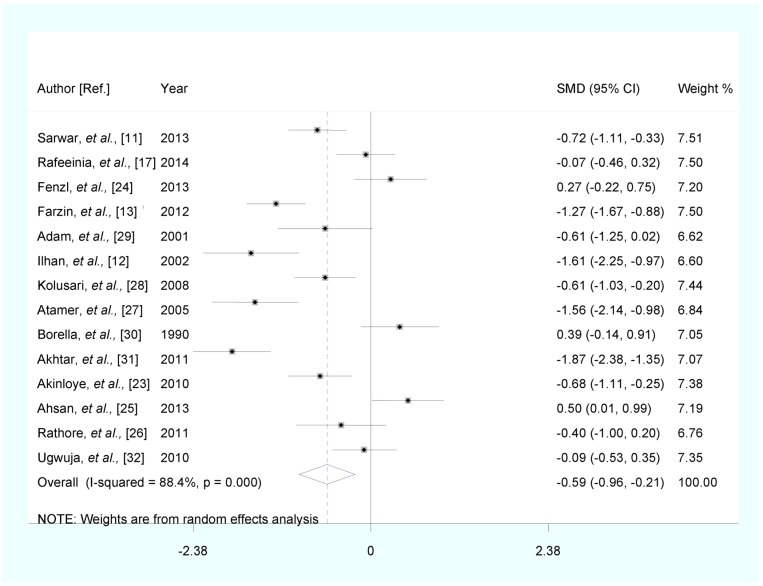
Forest plot of standard mean difference (SMD) with corresponding 95% confidence interval (CI) of studies on zinc levels in total preeclampsia (PE) and healthy pregnancy controls. The size of grey box is positively proportional to the weight assigned to each study, and horizontal lines represent the 95% CIs.

Fourteen articles reported the results for total PE compared with healthy pregnancy controls, PE patients have lower serum zinc level (SMD (95% CI): −0.587 (−0.963, −0.212), Z = 3.06, *p* for Z = 0.002; *I*^2^ = 88.4%; *p* for *I*^2^ < 0.0001) (Figure 2).

In subgroup analysis, the pooled SMD for studies conducted in Asia was −0.812 (95% CI: (−1.263, −0.362), *p* for Z < 0.0001). The pooled SMD was −0.636 (95% CI: (−1.131, −0.141), *p* for Z = 0.012) in studies involving fasting participants. When we stratified studies by different types of sample, the pooled SMD was −0.637 (95% CI: (−1.080, −0.195), *p* for Z = 0.005) in studies with zinc level measured in serum. In stratified analysis by status of healthy pregnancy controls matched by individual age, the pooled SMD was −0.634 (95% CI: (−1.213, −0.056), *p* for Z = 0.032). The pooled SMD was −0.678 (95% CI: (−1.325, −0.030), *p* for Z = 0.040) in stratified analysis by status of healthy pregnancy controls matched by individual gestational age. The results of subgroup analysis are shown in Table 2.

**Figure 3:**
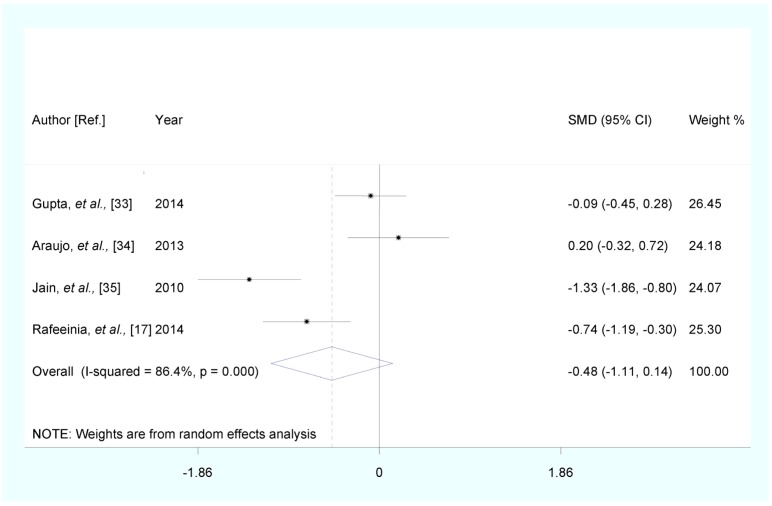
Forest plot of standard mean difference (SMD) with corresponding 95% CI of studies on zinc levels in mild PE and healthy pregnancy controls. The size of greybox is positively proportional to the weight assigned to each study, and horizontal lines represent the 95% CIs.

**Figure 4:**
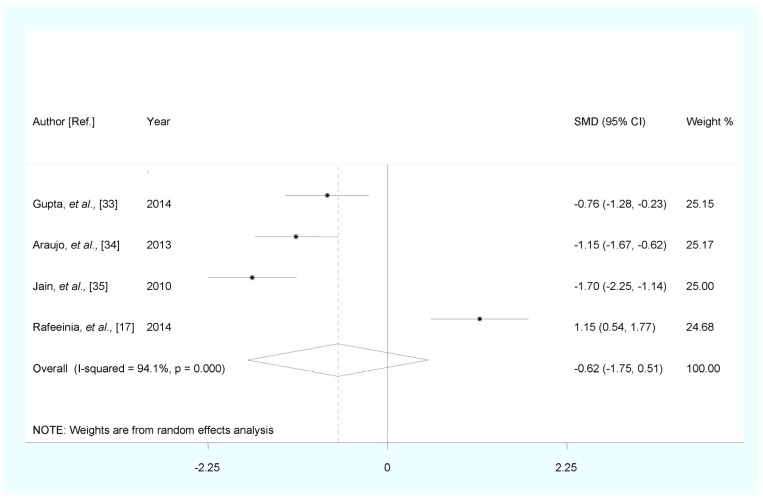
Forest plot of standard mean difference (SMD) with corresponding 95% confidence interval (CI) of studies on zinc levels in severe preeclampsia (PE) and healthy pregnancy controls. The size of grey box is positively proportional to the weight assigned to each study, and horizontal lines represent the 95% CIs.

Stratified analysis by PE disease severity showed that, compared with healthy pregnancy controls, the pooled SMD was −0.484 (95% CI: (−1.105, 0.137), Z = 1.53, *p* for Z = 0.126; *I*^2^ = 86.4%; *p* for *I*^2^ < 0.0001) for mild PE, and −0.618 (95% CI: (−1.750, 0.515), Z = 1.07, *p* for Z = 0.285; *I*^2^ = 94.1%; *p* for *I*^2^ < 0.0001) for severe PE (Figure 3 and Figure 4).

**Table 2:** Subgroup analyses of zinc level and preeclampsia (PE).

Subgroup	Number of Studies	SMD (95% CI)	Test of SMD = 0		Heterogeneity		Article Included
			Z	*p* for Z	*I*^2^	*p* for *I*^2^	
Continent							
Asia Europe Africa	10 2 2	−0.812 (−1.263, −0.362) 0.323 (−0.033, 0.678) −0.389 (−0.971, 0.194)	3.54 1.78 1.31	0.0001 0.075 0.191	88.4 0.0 72.2	0.0001 0.734 0.058	[11,12,13,17,25,26,27,28,29,31] [24,30] [23,32]
Sample type							
plasma serum	4 10	−0.460 (−1.246, 0.325) −0.637 (−1.080, −0.195)	1.15 2.82	0.251 0.005	87.7 89.4	0.0001 0.0001	[12,29,30,32] [11,13,17,23,24,25,26,27,28,31]
Fasting status							
yes no	8 6	−0.636 (−1.131, −0.141) −0.522 (−1.159, 0.115)	2.52 1.61	0.012 0.108	89.2 89.4	0.0001 0.0001	[11,12,13,17,24,27,28,30] [23,25,26,29,31,32]
Individual age match							
yes no	7 7	−0.634 (−1.213, −0.056) −0.540 (−1.060, −0.019)	2.15 2.03	0.032 0.042	89.9 88.2	0.0001 0.0001	[13,23,25,26,29,31,32] [11,12,17,24,27,28,30]
Individual gestational age match							
yes no	6 8	−0.678 (−1.325, −0.030) −0.516 (−0.983, −0.050)	2.05 2.17	0.040 0.030	91.5 86.0	0.0001 0.0001	[11,13,25,29,31,32] [12,17,23,24,26,27,28,30]

Abbreviations: SMD: standard mean difference; CI: confidence interval.

### 3.3. Meta-Regression

Strong evidence of heterogeneity among studies was found (Figure 2). However, the *P* values from univariate meta-regression analysis with the covariates of publication year, continent, sample type, fasting status of participants, individual age match, individual gestational age match and quality assessment were 0.930, 0.216, 0.710, 0.790, 0.832, 0.718 and 0.874, respectively. The results showed that no above-mentioned covariates conferred significant impact on between-study heterogeneity. The results of meta-regression are shown in Table S2.

### 3.4. Influence Analysis and Small-Study Effect Evaluation

**Figure 5:**
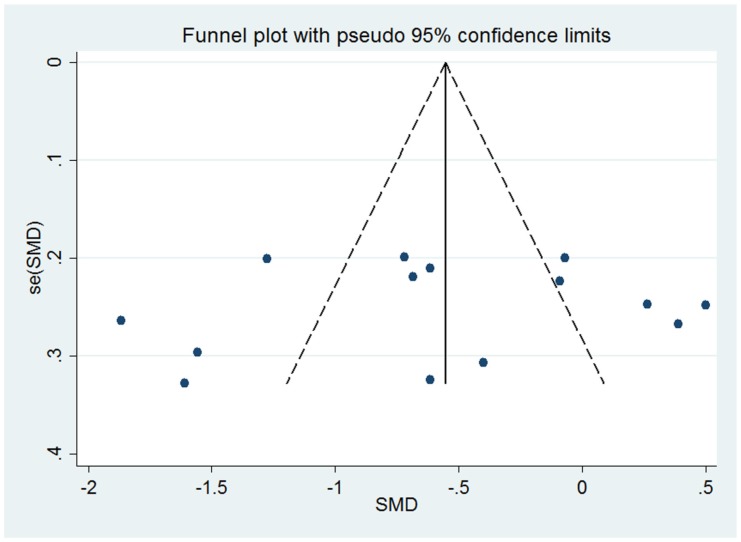
Funnel plot for the analysis of serum zinc level and preeclampsia (PE).

In influence analyses, we excluded 1 study at a time to assess the stability of the results. There was no significant change in the pooled SMD on excluding any of the studies (SMD lied between −0.671 and −0.488). This means no individual study had an excessive influence on the pooled effect between serum zinc level and PE. The visual inspection of the funnel plot was symmetrical (Figure 5). The Egger test showed no evidence of significant small-study effect for the analysis between serum zinc level and PE for all included studies (*p* = 0.621).

We also conducted the above-mentioned analysis with weighted mean difference (WMD). The results of pooled WMD were consistent with those of SMD. The details of pooled WMD are shown in Supplementary Table S3.

## 4. Discussion

Our meta-analysis contained 17 articles, including 725 healthy pregnancy controls and 700 PE patients. The result of 14 articles about total PE identified that serum zinc level in PE patients was significantly lower than that in healthy pregnancy controls. In subgroup analysis, the lower serum zinc level in PE patients compared with healthy pregnancy controls was observed in studies conducted in Asia, studies with zinc level measured in serum, and studies involving fasting participants. The SMD did not differ significantly between studies with healthy pregnancy controls matched by age (yes or no), and by gestational age (yes or no), respectively. The serum zinc levels were lower in the PE patients compared with healthy pregnant controls in studies conducted in Europe and Africa, studies with zinc level measured in plasma, and studies involving participants without fasting, but the results were not statistically significant. Our meta-analysis didn’t find significant difference between serum zinc level and mild PE or severe PE, which might be caused by limited number of included studies.

The mechanisms underlying the association between serum zinc level and PE are still not fully understood. One underlying explanation for our findings is that zinc can alleviate oxidative stress by increasing antioxidants or serving as essential substrates or cofactors for the adequate activation of antioxidant enzymes, such as superoxide dismutase (SOD) [11,48,49]. Zinc is a cofactor of the antioxidant enzyme SOD [49], thus its deficiency may lead to the decrease of SOD, which was associated with impairment of the cell antioxidant capacity and oxidant/antioxidant balance [10].

Cu-Zn SOD, the cytosolic form of enzyme, provide important antioxidant defense [50]. The deficiency of zinc has a negative effect on Cu-Zn SOD enzyme system [51]. Impaired Cu-Zn SOD activity contributes to the oxidative damage in the body which may worsen several disease states [52]. The main way that cells counteract free radical damage is by the increased expression of Cu-Zn SOD [34,53,54]. The decreased of Cu-Zn SOD activity may affect the scavenging of free radical and led to oxidative stress and lipid peroxidation [53,54]. Oxidative stress can induce apoptosis [55]. Exaggerated apoptosis may prevent supply of syncytiotrophoblast, promotesyncytial degeneration and release inflammatory mediators into the maternal circulation [56]. This would impair the placentation process and finally to diffuse maternal endothelial cell dysfunction [57]. All these may lead to the development of PE. Furthermore, Yousef *et al.* found that zinc deficiency can cause an increase in lipid peroxidation [58]. Zinc deficiency may cause the imbalance between lipid peroxides (LPO) and antioxidants by above mentioned ways, which might promote the occurrence and development of PE.

Between-study heterogeneity was found in our meta-analysis between serum zinc level and PE. We carried out meta-regression but did not find the covariates of publication year, continent, sample type, fasting status, age match and gestational age match as the important contributors to the between-study heterogeneity. Therefore, we speculated that the potential contributors to the conflicting results could be: (1) the included studies were different in blood sample handling methods and preservation methods; (2) the potential confounders adjusted in each study were diverse.

As a meta-analysis of published studies, our study has several strengths. First, the large numbers of participants allowed a much greater possibility of reaching reasonable conclusions and conducting subgroup analysis. Second, all included studies had accounted for potential confounders such as age and gestational age and additional factors, which can reduce the effects of confounding factors. Third, we adopted random effect model to calculate the pooled SMD between serum zinc level and PE; therefore, the results were more reasonable and convincing. Fourth, the Newcastle-Ottawa quality assessment scale was used to assess thestudy quality, and all studies met a quality score of 7 stars or more. The results indicated that the quality of original articles was generally good. Fifth, the physiological decrease of serum zinc level may occur during pregnancy; then original articles which we included in this meta-analysis chose healthy pregnancy women as controls. Sixth, serum zinc level was significantly lower in PE patients compared with healthy pregnancy controls in studies involving fasting participants. Fasting conditions can accurate reflect the metabolism of zinc. Seventh, the results of pooled SMD were consistent with those of pooled WMD, suggesting the results of our meta-analysis were credible. 

However, our study also has limitations. First, although the detection methods of serum zinc level were atomic absorption method, the testing instruments and testing conditions varied among studies; this may influence the detection results. Second, our meta-analysis didn’t find significant difference between serum zinc level and mild PE (*n* = 4) or severe PE (*n* = 4), which might be caused by the limited number of included studies and the limited sample size. Further research is needed to confirm the relation between serum zinc level and the disease severity of PE. Third, we were unable to explore the dose-response relationship between serum zinc level and PE because of the limitation of the data.

## 5. Conclusions

In summary, results from this meta-analysis showed that serum zinc level in PE patients was significantly lower than that in healthy pregnancy women. A moderate amount of zinc supplementation may reduce the incidence of PE, which needs to be confirmed.

## Supplementary Files

Supplementary File 1
