# Supplementary material for: The Relationship between Serum Zinc Level and Preeclampsia: A Meta-Analysis
Source: Nutrients. 2015 Sep 15;7(9):7806–20. doi: 10.3390/nu7095366 (PMC4586561; doi:10.3390/nu7095366)
Supplement: Supplementary File 1 [file nutrients-07-05366-s001.docx]

Supplementary Material

**Table S1.** Quality assessment of studies included in the meta-analysis.

| **ID** | **Author [Ref.]** | **Year** | **Country** | **Quality Assessment Criteria** | | | |
| --- | --- | --- | --- | --- | --- | --- | --- |
|  |  |  |  | **Selection** | **Comparability** | **Exposure** | **Overall Quality** |
|  |  |  |  | 1 2 3 4 | 5A 5B | 6 7 8 |  |
| 1 | Sarwar, M.S.; [1] | 2013 | case-control study | * * * | * * | * * * | 8 |
| 2 | Rafeeinia, A.; [2] | 2014 | cross-sectional study | * * * | * * | * * * | 8 |
| 3 | Fenzl, V.; [3] | 2013 | cross-sectional study | * * * | * * | * * * | 8 |
| 4 | Farzin, L.; [4] | 2012 | cross-sectional study | * * * | * * | * * * | 8 |
| 5 | Adam, B.; [5] | 2001 | cross-sectional study | * * * | * * | * * * | 8 |
| 6 | Ilhan, N.; [6] | 2002 | cross-sectional study | * * * | * * | * * * | 7 |
| 7 | Kolusari, A.; [7] | 2008 | cross-sectional study | * * * | * * | * * * | 8 |
| 8 | Atamer, Y.; [8] | 2005 | cross-sectional study | * * * | * * | * * * | 8 |
| 9 | Borella, P.; [9] | 1990 | cross-sectional study | * * * | * * | * * * | 7 |
| 10 | Akhtar, S.; [10] | 2011 | cross sectional study | * * * | * * | * * * | 8 |
| 11 | Akinloye, O.; [11] | 2010 | cross-sectional study | * * * | * * | * * * | 8 |
| 12 | Ahsan, T.; [12] | 2013 | cross-sectional study | * * * | * * | * * * | 8 |
| 13 | Rathore, S.; [13] | 2011 | cross-sectional study | * * * | * * | * * * | 7 |
| 14 | Ugwuja, E.I.; [14] | 2010 | cross-sectional study | * * * | * * | * * * | 8 |
| 15 | Gupta, S.; [15] | 2014 | case-control study | * * * | * * | * * * | 7 |
| 16 | AraújoBrito, J.; [16] | 2013 | case-control study | * * * | * * | * * * | 7 |
| 17 | Jain, S.; [17] | 2010 | cross-sectional study | * * * | * * | * * * | 8 |

The quality of studies was assessed by the Newcastle-Ottawa quality assessment scale.* 1, indicates cases independently validated; 2, cases are representative of population; 3, community controls; 4, controls have no history of blood pressure disease; 5A, study controls for age and gestational age; 5B, study controls for additional factor (s); 6, ascertainment of exposure by secure record; 7, same method of ascertainment used for cases and controls; and 8, non response rate the same for cases and controls.

**Table S2.** The results of univariate meta-regression analysis.

| **Covariates** | ***t*** | ***p*** |
| --- | --- | --- |
| Publication year | −0.09 | 0.930 |
| Continent | 1.31 | 0.216 |
| Sample type | 0.38 | 0.710 |
| Fasting status of participants | 0.27 | 0.790 |
| Age match | 0.22 | 0.832 |
| Gestational age match | 0.37 | 0.718 |
| Quality assessment | 0.16 | 0.874 |

**Table S3.** The pooled result of the meta-analysis with fixed effect model (FEM)
pooled weighted mean difference(WMD).

| **FEM Pooled WMD** | **WMD** | **95%CI** | | ***I*^2^ %** | ***p*** | **Test of WMD = 0** |
| --- | --- | --- | --- | --- | --- | --- |
| Pooled result of 14 studies | −0.053 | −0.079 | −0.027 | 95 | 0.0001 | Z = 3.96 *p* < 0.0001 |
| Subgroup | WMD | 95%CI | | I^2^ % | *P* | Test of WMD = 0 |
| Continent |  |  |  |  |  |  |
| Asia | −0.051 | −0.077 | −0.025 | 96.3 | 0.0001 | Z = 3.85 *p* < 0.0001 |
| Europe | 0.509 | −0.087 | 1.104 | 0 | 0.466 | Z = 1.67 *p* = 0.094 |
| Africa | −0.801 | −1.262 | −0.34 | 0 | 0.965 | Z = 3.40 *p* < 0.001 |
| Sample type |  |  |  |  |  |  |
| serum | −0.051 | −0.077 | −0.025 | 96 | 0.0001 | Z = 3.82 *p* < 0.0001 |
| plasma | −0.464 | −0.863 | −0.064 | 90.3 | 0.0001 | Z = 2.27 *p* = 0.023 |
| Fasting status |  |  |  |  |  |  |
| yes | −0.044 | −0.07 | −0.018 | 94.6 | 0.0001 | Z = 3.31 *p* < 0.001 |
| no | −0.967 | −1.24 | −0.694 | 94.2 | 0.0001 | Z = 6.93 *p* < 0.0001 |
| Age match |  |  |  |  |  |  |
| yes | −1.143 | −1.408 | −0.879 | 94.6 | 0.0001 | Z = 8.48 *p* < 0.0001 |
| no | −0.042 | −0.068 | −0.016 | 92.6 | 0.0001 | Z = 3.14 *p* = 0.002 |
| Gestational age match |  |  |  |  |  |  |
| yes | −1.375 | −1.696 | −1.053 | 95.5 | 0.0001 | Z = 8.37 *p* < 0.0001 |
| no | −0.044 | −0.07 | −0.018 | 91.3 | 0.0001 | Z = 3.29 *p* < 0.001 |
| **Illness Severity** | **WMD** | **95%CI** | | ***I*^2^ %** | ***p*** | **Test of WMD = 0** |
| sever group | −1.075 | −1.434 | −0.715 | 95.8 | 0.0001 | Z = 5.86 *p* = 0.0001 |
| mild group | −0.505 | −0.735 | −0.275 | 90.2 | 0.0001 | Z = 4.30 *p* = 0.0001 |

Abbreviations: FEM: fixed effect model; WMD: weighted mean difference; CI: confidence interval.

References

1. Sarwar, M.S.; Ahmed, S.; Ullah, M.S.; Kabir, H.; Rahman, G.K.; Hasnat, A.; Islam, M.S. Comparative study of serum zinc, copper, manganese, and iron in preeclamptic pregnant women. *Biol. Trace Elem. Res.* **2013**, *154*, 14–20.
2. Rafeeinia, A.; Tabandeh, A.; Khajeniazi, S.; Marjani, A.J. Serum copper, zinc and lipid peroxidation in pregnant women with preeclampsia in Gorgan. *Open Biochem. J.* **2014**, *8*, 83–88.
3. Fenzl, V.; Flegar-Mestric, Z.; Perkov, S.; Andrišić, L.; Tatzber, F.; Žarković, N.; Duić, Ž. Trace elements and oxidative stress in hypertensive disorders of pregnancy. *Arch. Gynecol. Obstet.* **2013**, *287*, 19–24.
4. Farzin, L.; Sajadi, F. Comparison of serum trace element levels in patients with or without
   pre-eclampsia. *J. Res. Med. Sci.* **2012**, *17*, 938–941.
5. Adam, B.; Malatyalioqle, E.; Alvur, M.; Talu, C. Magnesium, zinc and iron levels in pre-eclampsia. *J. Matern. Fetal Med.* **2001**, *10*, 246–250.
6. Ilhan, N.; Simsek, M. The changes of trace elements, malondialdehyde levels and superoxide dismutase activities in pregnancy with or without preeclampsia. *Clin. Biochem.* **2002**, *35*, 393–397.
7. Kolusari, A.; Kurdoqlu, M.; Yildizhan, R.; Adali, E.; Edirne, T.; Cebi, A.; Demir, H.; Yoruk, I.H. Catalase activity, serum trace element and heavy metal concentrations, and vitamin A, D and E levels in pre-eclampsia. *J. Int. Med. Res.* **2008**, *36*, 1335–1341.
8. Atamer, Y.; Kocyigit, Y.; Yokus, B.; Atamer, A.; Erden, A.C. Lipid peroxidation, antioxidant defense, status of trace metals and leptin levels in preeclampsia. *Eur. J. Obstet. Gynecol. Reprod. Biol.* **2005**, *119*, 60–66.
9. Borella, P.; Szilagyi, A.; Than, G.; Csaba, I.; Giardino, A.; Facchinetti, F. Maternal plasma concentrations of magnesium, calcium, zinc and copper in normal and pathological pregnancies. *Sci. Total Environ.* **1990**, *99*, 67–76.
10. Akhtar, S.; Begum, S.; Ferdousi, S. Calcium and zinc deficiency in preeclamptic women.
    *J. Bangladesh Soc. Physiol.* **2011**, *6*, 94–99.
11. Akinloye, O.; Oyewale, O.J.; Oguntibeju, O.O. Evaluation of trace elements in pregnant women with pre-eclampsia. *Afr. J. Biotechnol.* **2010**, *9*, 5196–5202.
12. Ahsan, T.; Banu, S.; Nahar, Q.; Ahsan, M.; Khan, M.N.; Islam, S.N. Serum trace elements levels in preeclampsia and eclampsia: Correlation with the pregnancy disorder. *Biol. Trace Elem. Res.* **2013**, *152*, 327–332.
13. Rathore, S.; Gupta, A.; Batra, H.S.; Rathore, R. Comparative study of trace elements and serum ceruloplasmin level in normal and pre-eclamptic pregnancies with their cord blood. *Biol. Trace Elem. Res.* **2011**, *22*, 207–210.
14. Ugwuja, E.I.; Ejikeme, B.N.; Ugwu, N.C.; Obeka, N.C.; Akubugwo, E.I.; Obidoa, O. Comparison of plasma copper, iron and zinc levels in hypertensive and non-hypertensive pregnant women in Abakaliki, south Eastern Nigeria. *Pak. J. Nutr.* **2010**, *9*, 1136–1140.
15. Gupta, S.; Jain, N.P.; Avasthi, K.; Wander, G.S. Plasma and erythrocyte zinc in pre-eclampsia and its correlation with foetal outcome. *J. Assoc. Physicians India* **2014**, *62*, 306–310.
16. Araujo Brito, J.; do Nascimento Marreiro, D.; Moita Neto, J.M.; Michelle Costa e Silva, D.; Gonçalves de Sousa Almondes, K.; Valadares Neto Jde, D.; do Nascimento Nogueira, N. Enzyme activity of superoxide dismutase and zincemia in women with preeclampsia. *Nutr. Hosp.* **2013**, *28*, 486–490.
17. Jain, S.; Sharma, P.; Kulshreshtha, S.; Mohan, G.; Singh, S. The role of calcium, magnesium, and zinc in pre-eclampsia. *Biol. Trace Elem. Res.* **2010**, *133*, 162–170.

© 2015 by the authors; licensee MDPI, Basel, Switzerland. This article is an open access article distributed under the terms and conditions of the Creative Commons Attribution license (http://creativecommons.org/licenses/by/4.0/).
